# Learning Shapes Spontaneous Activity Itinerating over Memorized States

**DOI:** 10.1371/journal.pone.0017432

**Published:** 2011-03-08

**Authors:** Tomoki Kurikawa, Kunihiko Kaneko

**Affiliations:** Department of Basic Science, University of Tokyo, Tokyo, Japan; Indiana University, United States of America

## Abstract

Learning is a process that helps create neural dynamical systems so that an appropriate output pattern is generated for a given input. Often, such a memory is considered to be included in one of the attractors in neural dynamical systems, depending on the initial neural state specified by an input. Neither neural activities observed in the absence of inputs nor changes caused in the neural activity when an input is provided were studied extensively in the past. However, recent experimental studies have reported existence of structured spontaneous neural activity and its changes when an input is provided. With this background, we propose that memory recall occurs when the spontaneous neural activity changes to an appropriate output activity upon the application of an input, and this phenomenon is known as bifurcation in the dynamical systems theory. We introduce a reinforcement-learning-based layered neural network model with two synaptic time scales; in this network, I/O relations are successively memorized when the difference between the time scales is appropriate. After the learning process is complete, the neural dynamics are shaped so that it changes appropriately with each input. As the number of memorized patterns is increased, the generated spontaneous neural activity after learning shows itineration over the previously learned output patterns. This theoretical finding also shows remarkable agreement with recent experimental reports, where spontaneous neural activity in the visual cortex without stimuli itinerate over evoked patterns by previously applied signals. Our results suggest that itinerant spontaneous activity can be a natural outcome of successive learning of several patterns, and it facilitates bifurcation of the network when an input is provided.

## Introduction

One of the most important features of the brain is the ability to learn and regenerate an appropriate response to external stimuli. By modification of the synaptic strength, output responses to input stimuli are memorized. Accordingly, these input-output (I/O) mappings are embedded in synaptic structure. A wide variety of neural network models have been proposed to study how a synaptic structure is formed for memorizing the given I/O mappings. In most of the previous studies on unsupervised learning [1]–[3], inputs were supplied as the initial states for neural activity, whose temporal evolution results in the generation of the desired outputs. Similarly, in supervised learning with multi-layer neural networks [4], inputs are provided as the initial states to an input layer, and the neural activity in the output layer is determined on the basis of the inputs. In this manner, an input determines the initial states of the system, while an output is given by an attractor of the neural activity dynamics. Here, the learning process changes the dynamical system so that the postulated output is generated by the attractor to which the neural activity is attracted under the initial conditions. Each output pattern is thus memorized as an attractor, and this process is often referred to as “memories as attractors.”

In these studies, the input is specified only as the initial neural activity. Hence, neural activity dynamics cannot be determined accurately in the absence of inputs since the initial values for neural activity are chosen on the basis of the inputs. However, many studies have indicated that for understanding the functioning of the brain, it is important to induce neural activity in the absence of inputs. In particular, recent studies have shown that in the brain, structured neural activity is observed even in the absence of external stimuli [5]; such an activity is termed “spontaneous activity.” After input is provided, this spontaneous activity is modified so that an appropriate output response is generated. In recent experiments [6] on an olfactory system, the neural dynamics have been studied in the presence and absence of odor stimuli. Steady states of the neural activity, which are different from the rest state, are generated for different odor stimuli; the neural activity returns to the rest state upon removal of the external stimulus. Hence, an input modulates spontaneous activity to generate an output rather than determines the neural state as the initial state. These observations strongly indicate a novel I/O representation in the neural activity dynamics, which also includes spontaneous activity.

In this paper, we propose a novel viewpoint of the memory of I/O mapping, in order to verify the aforementioned postulate. For this purpose, we present the following questions: Can we construct an appropriate neural network model to demonstrate the learning process under biologically plausible assumptions? If so, under what conditions would learning be possible? Then, what types of spontaneous activity, which can change the desired output depending on the corresponding input, are shaped? What changes in the neural activity can bring about the output when an input is provided?

In the present study, we find the answers to these questions by adopting a layered neural network model for reinforcement learning along with multiple time scales for synaptic plasticity and the associative reward-penalty algorithm (ARP) [7] [8]. We demonstrate that the proposed model can memorize the maximum number of I/O mappings when the time scales of the plasticity of the forward and backward synapses satisfy a certain condition.

In our theoretical framework, an output for a given input is represented by an attractor of the neural dynamics in the presence of the input; this attractor may differ from that in the absence of the input. The nature of the attractor changes with the input and such a qualitative change in the attractor with the parameters of a dynamical system is referred to as “bifurcation” in the dynamical systems theory. Hence, the input-induced change in the attractor is represented as bifurcation in the dynamical systems theory. In other words, an input can be considered a bifurcation parameter for neural activity dynamics. Dynamical systems are generally represented by the flow structure in the state space, and hence this flow structure changes with the input so that a state that represents a given target output is generated. In this dynamical-system perspective, learning helps in the formation of an appropriate flow structure through bifurcations caused by changes in the strength of the applied input. When an I/O mapping is memorized, the neural dynamics undergo bifurcation, and the spontaneous dynamics attractor is converted into an attractor representing the desired output for a given input. When the learning process progresses, the neural dynamics are modified so that the aforementioned “bifurcation” occurs.

We show that in the absence of an input, the neural dynamics itinerate over several states corresponding to each of the memorized output patterns after many targets have been learned. This theoretical finding is in remarkable agreement with recent experimental report [9]. This report states that in the absence of stimuli, the spontaneous neural activity in the visual cortex itinerates over patterns evoked by the visual signals. We analyze how the flow structure of the neural network is shaped by the learning process and discuss the possible relationship between our results and recent experimental observations of the external-stimuli-induced modification of spontaneous activity.

## Methods

### Architecture of Our Model

We construct a neural network model for learning, on the basis of the following two conditions that satisfy the biological requirements for the normal functioning of the brain: (i) different error information for different individual neuron should not be required. In other words, individual error information is used commonly to all neurons. For example, in the error back-propagation algorithm [4], one of the most popular learning algorithms for neural networks, information corresponding to each of the output neurons is required. In the case of biological learning with a neural system, however, it is difficult to transmit the specified information to each neuron. (ii) I/O mappings should be learned one by one sequentially, i.e., a new I/O mapping should be learned only after the previous mapping has been learned while preserving the previously learned mappings. In contrast, in most learning algorithms for neural networks, many mappings are simultaneously and iteratively learned by gradually changing the synaptic strength until all the mappings are memorized.

**Figure 1:**
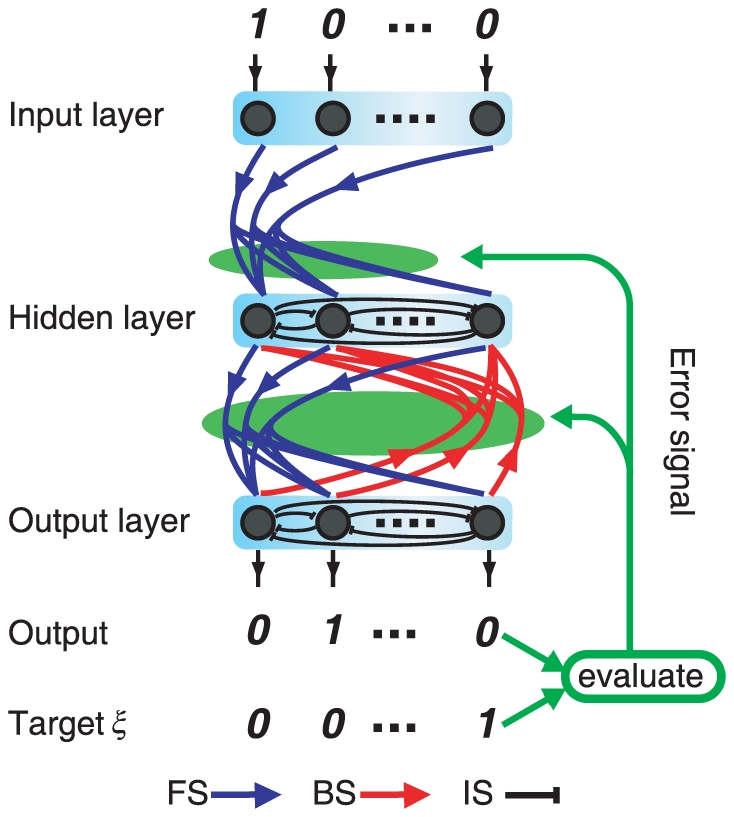
Schematic representation of the network architecture of our model. FS (BS) represents the interlayer forward (backward) excitatory synapse, and IS represents the mutually inhibitory intralayer synapse. Error signal represents the difference between an output and a target pattern and regulates the plasticity of all subsequent FSs and BSs (Eq.3) (green arrows and ellipses).

In order to satisfy the above-mentioned conditions, we introduce a layered network model consisting of input, hidden, and output layers along with ARP algorithm for reinforcement learning (Fig. 1) [7] [8]. In this model, several I/O mappings are learned one by one with only a single error signal that is defined as the distance between the activity pattern of the output neurons and a given target pattern. During the learning process, the plasticity of the synaptic strength varies with the magnitude of the error signal, in accordance with the Hebbian and anti-Hebbian rules.

In particular, we adopt the following model with 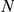 neurons in each layer. Three types of synapses are considered: excitatory forward synapses (FSs), excitatory backward synapses (BSs), and mutually inhibitory intralayer synapses (ISs). FSs connect the neurons in the input layer to those in the hidden layer and the neurons in the hidden layer to those in the output layer. BSs connect the neurons in the output layer to those in the hidden layer, while ISs connect the neurons within a given layer (hidden or output layer). This architecture is similar to that of a simple recurrent network (SRN) [10] [11]. As opposed to the study on temporal evolution of I/O mappings in an SRN, the present study deals with the shaping of neural dynamics in the presence and absence of the input through learning process.

### Neural Dynamics

The neural activity in the input layer is fixed at an input pattern 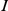, an 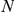-dimensional vector whose element takes the value 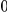 or 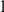 and the magnitude of the vector is 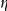 (Eq.1). We use the rate-coding neuron model for the neural activities in the other layers (Eq.2), because this is simple but general model that has been used widely and is well suited for dynamical-system analysis.
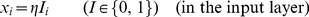
(1)

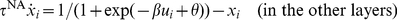
(2)where 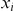 is the firing rate of neuron 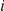, and 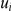 is the input current applied to the neuron 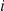. The input current is given by 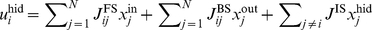 for the neurons in the hidden layer and 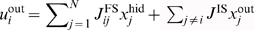 for the neurons in the output layer. Here, 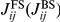 is the strength of the forward (backward) synapse from a presynaptic neuron 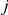 to a postsynaptic neuron 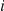. 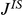 is the strength parameter for the mutually inhibiting IS; this parameter assumes a fixed and identical value for all ISs (set at −1.0) except for self-connected synapses (set at 0.0). 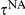 is a time scale of neural activities and is set to 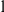 in the present case. We also analyze the dependency of memory capacity on the relationship among the three time scales in the system: the time scale for neural dynamics and the time scale for the plasticity of FSs and BSs. The other parameters are set as follows: 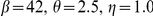, and 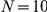. Because of the competition through the ISs, only one (or very few) neuron(s) in each layer is (are) excited, and this results in a sparse neural activity pattern. Once an input pattern is given as a boundary condition, the evolution of neural activities in the hidden and output layers are determined by the above-defined dynamical systems. Hence, each input modifies the flow structure of neural dynamics composed of neurons in the hidden and output layers, resulting in modifying the neural dynamics on the basis of this flow structure.

### Synaptic Plasticity

Synaptic plasticity is necessary for learning in a neural network. As mentioned earlier in the text, we maintain the strength of the ISs constant for simplicity and vary the strengths of the FSs and BSs. For each input pattern defined above, we prescribe a target pattern 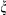 as an 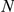-dimensional vector whose element takes the value 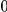 or 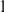, and choose sparse patterns in which only one neuron is activated as inputs and targets. In the learning task, the neural activity in the output layer is described as the 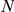-dimensional vector 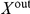, and the error 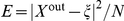 is minimized. We adopt two schemes for synaptic plasticity: multiple time scales and the ARP algorithm for reinforcement learning [7] [8]. First, the time scale of the plasticity of FSs (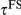) is different from that of the plasticity of BSs (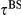). Second, the synapse pattern that generates the target output is strengthened, in accordance with the Hebbian rule; otherwise, it is weakened, as per the anti-Hebbian rule. In accordance with the ARP, we assume that the synaptic dynamics depend on the activities of the pre- and postsynaptic neurons as well as on 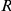 determined from the error signal 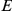, as
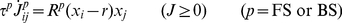
(3)

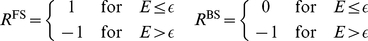
(4)


Here, 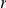 is the spontaneous firing rate (set at 0.1) and 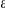 is set at 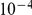. The sign of 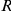 changes with the magnitude of the error signal 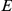 between the output pattern and the target pattern. When the output pattern is close to the target pattern, i.e., 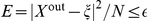, the synaptic plasticity follows the Hebbian rule, which is derived by substituting 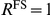 and 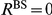 in (Eq.3). This plasticity stabilizes the ongoing neural activity pattern. Note that during this stabilization process, only the strength of the FS varies, and hence, memories of the I/O mappings are embedded in the FSs. In contrast, when the output pattern is distant from the target pattern, i.e., 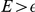, the synaptic plasticity follows the so-called anti-Hebbian rule, and hence, the ongoing neural activity pattern is destabilized. Note that with the above form 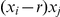, the synapse shows negligible changes when its pre-synaptic neuron 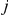 is in a low-firing state. In our model, we require only a single error term for all neurons; this is in strong contrast to error back-propagation, which requires the computation of a large number of error terms, i.e., as many error terms as the output neurons. Brief and the preliminary report of this model is given in the proceeding [12].

In most neural network studies, only two time scales are considered: one for neural activities and the other for synaptic plasticity. In this study, we consider a variety of time scales for synaptic plasticity and introduce two time scales for the plasticity of the FSs and BSs. As will be shown later, I/O mappings are successfully memorized when the difference between the time scales is appropriate.

## Results

### Neural Dynamics in the Learning Process

**Figure 2:**
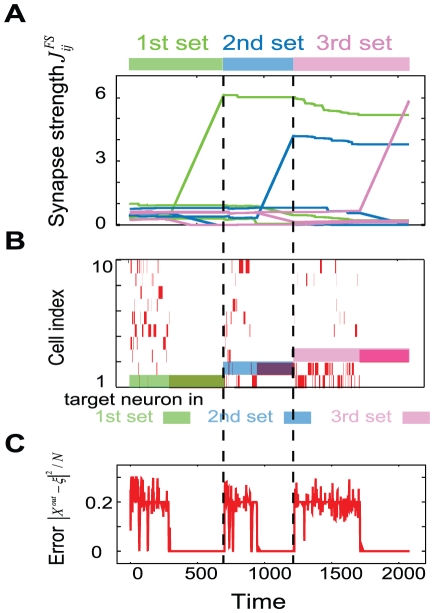
Dynamics during the learning of three input-output (I/O) mappings. I/O mappings are learned in the search phase by the anti-Hebbian rule (0
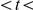
300, 700
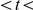
950, and 1200
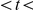
1700) and in the stabilization phase by the Hebbian rule (300
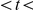
700, 950
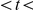
1200, and 1700
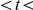
2100). Color bars on the top of figures and above the time series in B) represent each set of input/target patterns. A) Dynamics of some FSs are shown. These lines represent FSs from neurons in hidden layer to the neurons to be activated in targets in output layer. These neurons are referred to as the target neurons. In particular, each color (green, blue and red) represents FSs to each target neuron (1st, 2nd and 3rd target) respectively. Three lines increasing rapidly are FSs from activated neurons in hidden layer to the target neurons. B) Raster plot of neurons in the output layer is shown. The ordinate shows the index of the neurons in the output layer. Red bar represents the high activity of each neuron (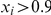). Blue (green) bar behind the Raster plot indicates the output corresponding to the first (second) target. C) Time series of the amplitude of the error signal between the output and target patterns. Distance *d* between the output pattern 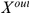 and the target pattern 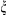 by the normalized Euclidean norm (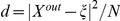) as a function of time is plotted.

We show that our model can learn I/O mappings based on our perspective. An example of the learning process is shown; the time series of the strength of some synapses, that of the neural activities in the output layer and that of the error signal during the learning process are shown in Figure 2. As the initial conditions for the network, we set 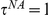, 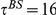, and 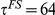 and assign the synaptic strength a random value with a uniform distribution between 0 and 1, except in the case of the ISs.

When the error is large (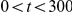 in Fig. 2), the neural dynamics itinerate between different patterns since the present neural activity becomes unstable as per the anti-Hebbian rule. The target pattern is searched during this itineration. We term this period “search phase” in what follows. At 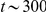, the magnitude of the error reduces to a sufficient extent, i.e., the output dynamics of the neural activity are within the neighborhood 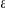 of the target, where the synaptic plasticity changes from the Hebbian rule to the anti-Hebbian rule. Once this occurs, the neural activity is stabilized as per the Hebbian rule (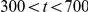), and the output activity remains close to the target, in accordance with the Hebbian rule; because of this, the synapses between active neurons are continuously strengthened until a new target is generated (Fig. 2). This period is called “stabilization phase.” At 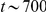, we switch the input and the corresponding target patterns to generate new input-target pairs. Then, the distance between the output pattern and the target pattern increases again, and therefore, the search process progresses according to the anti-Hebbian rule (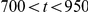) until the stabilization phase is initiated as per the Hebbian rule at 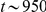 when the output activity is close to the target. Furthermore, at 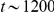, we switch the input and the target pattern to generate the third input-garget pairs and the learning process progresses in the same manner as mentioned above. In this manner, the neural activity can be made to approach the target and the target learning can be achieved by making the synaptic plasticity alternately anti-Hebbian and Hebbian, depending on the error. Note that in this phase, the target is learned in the flow structure in the presence of the corresponding input, but not in the absence of the input, i.e., an attractor in the presence of an input, but not an attractor in the absence of an input, is formed by the learning process.

### Memory Capacity through the Learning Process

We discuss the memory capacity through the learning process. For specificity, we use the following procedure for counting the number of memories: After each learning step, which consists of a search phase and a stabilization phase, we apply each of the inputs learned so far and check whether the output pattern matches the corresponding target pattern for most of the initial neural activity values, by fixing the synaptic strength. Here, “most” means that the fraction of the initial values reaching the target pattern is greater than one-half. (If this threshold value is changed, the number of memories is modified. However, the results below are not essentially changed, as long as it is neither too smaller nor too large.)

**Figure 3:**
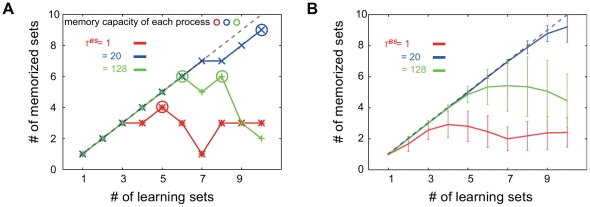
The number of memories through the learning process. The number of memorized sets at each learning step is plotted for three different values of 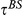, by fixing 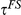 at 64. Red, green, and blue lines represent the number of memorized sets for 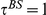 (identical to that 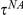), 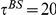 (maximum memory capacity), and 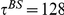, (larger than at 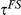), respectively. Dotted line represents the maximum possible number of memorized sets, i.e., the number of all learned I/O relations. A) Number of memorized sets at a single learning process. Memory capacity at each value of 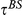 is defined by the maximum number of memorized sets in the learning process, as shown by colored circles. B) Number of memorized sets averaged over learning processes. The number of learned sets is calculated by averaging over 100 learning processes for each value of 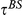. Error bars indicate the standard deviations.

In other words, when the I/O mapping is memorized, the neural activity comes close to the target by applying the input, irrespective of the state before applying the input. The number of memorized targets increases with the sequence of learning steps (i.e., the number of I/O mappings provided) and reaches a saturation value (or decreases) because of the loss of the earlier memory (Fig. 3). Then, the memory capacity is determined from the maximum number of memorized sets in the entire learning process.

In our model, the I/O mappings to be learned are provided sequentially, and the learning process where each mapping is provided only once is mainly analyzed. However, it is also possible to learn the mappings repeatedly. In our model, the synaptic strength is not limited, and therefore, the magnitude of synaptic connections between the active neurons would increase unlimitedly as a result of repeated learning. In the results shown below, we change the initial condition for the synaptic strength to 0, except in the case of the ISs, before the commencement of the learning process in order to prevent the initial network from being infected.

### Dependence of Memory Capacity on Timescales

**Figure 4:**
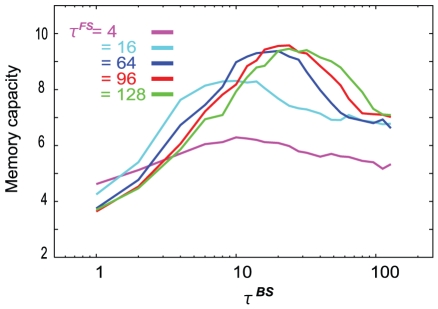
Memory capacity as a function of 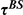 for various values of 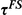 Memory capacity curves as a function of 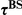 are shown (See text for the definition of capacity). The other time scale, 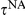, is fixed at a unit value. Memory capacity shows a peak when 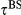 satisfies the condition 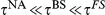 If 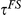 is sufficiently large, our model can memorize almost all learned sets (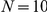) at the time scale satisfying this condition. Here, 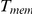 at each 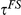 is scaled by 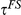, i.e., 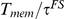 is constant. Computed from the average over 100 learning processes for each 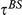.

**Figure 5:**
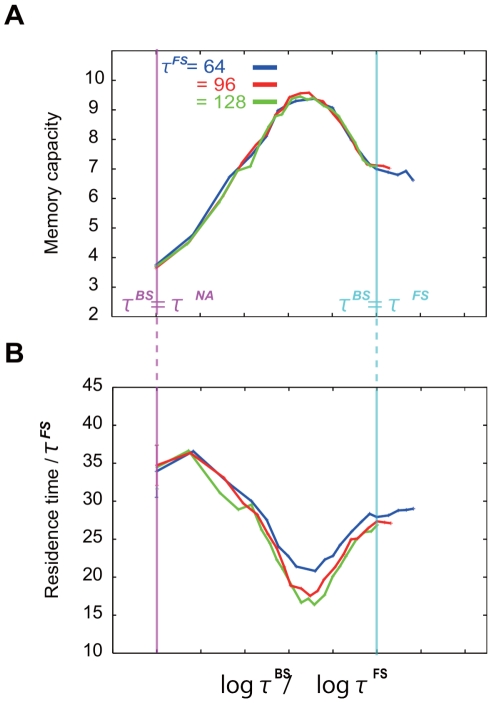
Scaled capacity curve and residence time curve. Scaled capacity curve (i) and residence time curve (ii) when 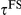 is large (64, 96, and 128) are plotted. Capacity and residence time are plotted as functions of the ratio of the logarithms of 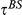 and 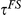 and not as functions of the logarithm of 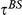. Here, the residence time is scaled by 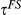. Vertical pink and blue lines represent the values of 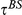 satisfying the conditions 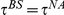 and 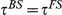 respectively. All capacity curves show a peak at the same value of 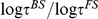, where the residence time takes the minimum value. Here the residence time is defined as the time at which the output neural pattern is closer to the given target than the threshold distance. To be specific, we set the threshold distance between the output and target at 0.9.

As has been mentioned before, there are three time scales in our model: 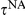 for changes in the neural activity and 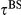 and 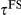 for the plasticity of the BSs and FSs, respectively. We analyze the dependence of the memory capacity on 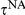, 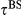, and 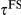. Specifically, we compute the capacity by fixing 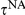 and 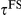 and study the dependence of the memory capacity on 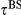. In Figure 4, the memory capacity is plotted as a function of 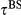 for various values of 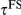. We confirm that the memory capacity is small for any 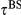, unless 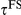 is sufficiently larger than 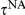; this is because there is no method for preserving the information about previously learned patterns that are embedded in the FSs. Thus, we focus on the capacity in the case of 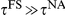 in Figure 5. Interestingly, each capacity curve in the plot shows a peak when the time scale satisfies the condition 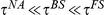, where the capacity is 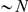, which is equal to the number of neurons in each layer. Since the present model adopts the sparse coding principle, 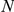 is the maximum possible memory capacity. When 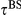 approaches 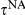 or 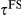, the capacity is considerably smaller than the maximum capacity. Furthermore, after scaling 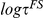, the capacity curves nearly overlap with one another. By this scaling, the position of the peak is independent of 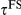 unless 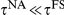.

In the search phase, the synaptic plasticity in the learning process modifies the flow structure in the phase space of the neural state so that the neural activity searches the target by itinerating various patterns including the learned target pattern. We focus on when the output activity come close to one of the previously learned target patterns in the search phase. Since this pattern differs from the current target pattern, the flow structure attracting to the previous target pattern may be destroyed by the synaptic plasticity, as stated by the anti-Hebbian rule. Thus, the flow structure in the phase space of the neural state that supports the attraction to the previously memorized pattern may be destroyed. In general, the longer the output pattern stays close to a state corresponding to a previously learned pattern, the stronger is the destabilization of the attractive flow to the state. Hence, the degree of destabilization of the previous memory is expected to increase with the residence time of the pattern in the corresponding state. Indeed, as shown in Figure 5, the residence time, when plotted as a function of 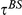, decreases to a minimum when 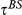 corresponds to the maximum memory capacity and increases as 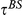 approaches either 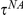 or 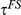. This trend is consistent with the dependence of the memory capacity on 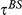.

Now we discuss the condition 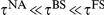. Either, 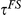 or 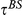 determines the time scale for the change in the flow structure itself, whereas 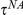 determines that of the neural dynamics of a certain fixed flow structure. Moreover, because the search for the target is based on the change in the flow structure, as per the anti-Hebbian rule, the time scale of the search phase is effectively determined by 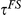 or 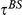. If 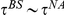, the flow structure in the phase space is modified during the neural activity change, and hence, the approach the target pattern is often hindered. Indeed, the search for a new target pattern takes a longer time to complete as 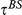 approaches 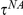, and the residence time in the previously memorized patterns increases. This results in a decrease in the memory capacity.

On the other hand, the time scale of the memory decay is determined by 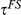 because the memory information is embedded in the FSs (Eq.4). Here, 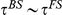 indicates that the time scale of the search phase is equal to that of the memory decay; during the search for the target, the memory of the previously learned mappings is destroyed. Thus, the condition 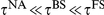 must be satisfied for successive learning.

**Figure 6:**
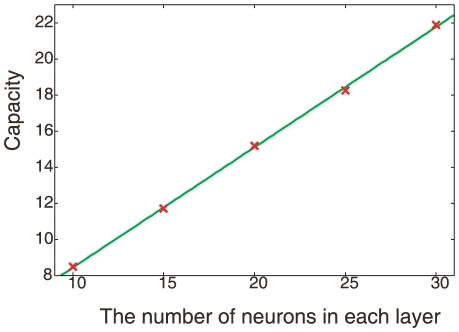
Capacity dependence on N. Capacities for various numbers of neurons 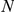 are plotted. Each capacity is measured after learning the maximum number of I/O mappings (i.e., the number of neurons in each layer 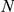). The time scales are 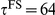 and 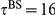, which is independent of 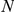, while 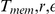 are in proportion to 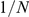.

Here, we briefly discuss the dependence of memory capacity on the number of neurons 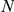. As long as the sparse firing in the hidden layer is satisfied, the maximal capacity is expected to be approximately 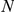, if the timescale relationship between 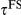 and 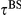 is fine-tuned on the basis of 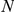. Even without such fine-tuning, the capacity increases roughly with 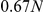 when 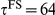 and 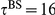 (some parameters are scaled by 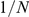; details can be found in the caption of Fig. 6). Hence, a rather high capacity is achieved. However, with an increase in 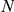, the learning time increases, as the phase space in neural activity to be searched increases with 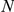. In fact, when 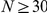 the search time is so long that the memory is often destroyed during the search phase, unless the parameters 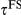 and 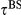 are fine-tuned. Here after, we fix 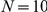 to discuss the neural activity dynamics.

### Shaping of Spontaneous Activity by Learning

Now, we analyze how the networks memorize the I/O mappings, by imposing the condition 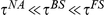. We set 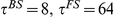, and 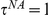, under which conditions the capacity is close to the maximum possible value.

**Figure 7:**
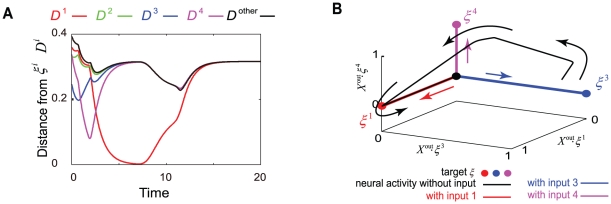
Temporal evolution of neural activity in the case of fixed point. The spontaneous neural dynamics and the evoked one after four I/O mappings are learned are plotted. A) The time courses of the distance between the neural activity of the output and each target are plotted. Here, only the time courses representing the neural dynamics in the absence of input are plotted. The distance is defined as 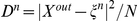, The curves in different colors represent the distance from each target, while the black curve shows the distance from an unlearned target pattern, for reference. B) Neural activity in the output layer is represented as a point in the three-dimensional space and is projected from the 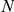-dimensional space consisting of neural activities in the output layer by obtaining the product of the output activity and the target pattern(s). Each axis represents the product of neural activity and the corresponding target pattern, defined by axis1 = 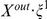, axis2 = 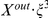, and axis3 = 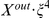. The orbits of neural activities after learning I/O mappings are plotted. The black line and circle represent a transient trajectory and a fixed-point attractor in the absence of inputs, respectively. The colored (red, green, and blue) lines and circles represent the trajectories and fixed-point attractors in the presence of inputs, respectively, with each color indicating the corresponding input. In the absence of an input, the neural activity once approaches some fixed point corresponding to the given target outputs and then departs, before finally converging to the fixed-point attractor. Upon the application of an input, the fixed-point attractor becomes unstable, and hence, the neural activity is attracted to a new fixed point, thereby giving rise to a target output corresponding to the input.

**Figure 8:**
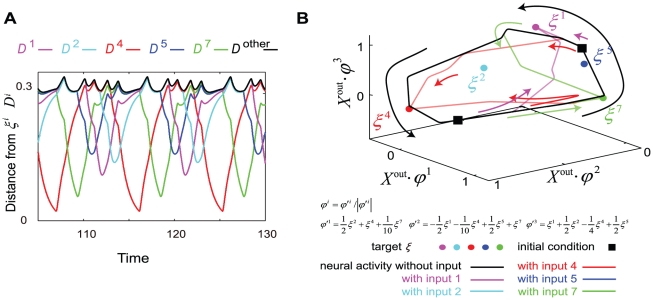
Temporal evolution of neural activity when the attractor without inputs is a limit cycle. The spontaneous neural dynamics and the evoked one after learning eight I/O mappings are plotted, same as Fig. 7. A) The time courses of the distance between the neural activity of the output and each target are plotted. Notations are same as Fig. 7 A. The time courses after transient time are plotted for focusing on limit-cycle attractor. B) Neural activity in the output layer is represented as a locus in a three-dimensional space, projected from the 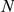-dimensional space consisting of neural activities in the output layer, by obtaining the product of the output activity and the combined target pattern(s). Each axis represents the product of the neural activity and the corresponding combined target patterns 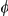, defined in the figure. Plotted by using the same notations used in Fig. 7. The black curve is not a transient orbit but a limit cycle in the absence of inputs. The colored curves represent the transient trajectories when inputs are applied under two initial conditions (black squares), which are not the initial conditions for the limit cycle. In the presence of an input, the limit-cycle attractor collapses, and the neural activities reach fixed points, giving rise to the corresponding target outputs. These fixed points are represented by circles in different colors, while transient trajectories from only two initial points are shown here. However, trajectories from all points on the limit-cycle reach the corresponding target with the application of a given input. On the other hand, the limit cycle attractor in the absence of inputs approaches and deviates from the points matching the targets, which are fixed points in the presence of inputs as mentioned above. The bifurcation from the spontaneous limit-cycle attractor to the evoked fixed-point attractor is plotted in detail in Fig. 12.

**Figure 9:**
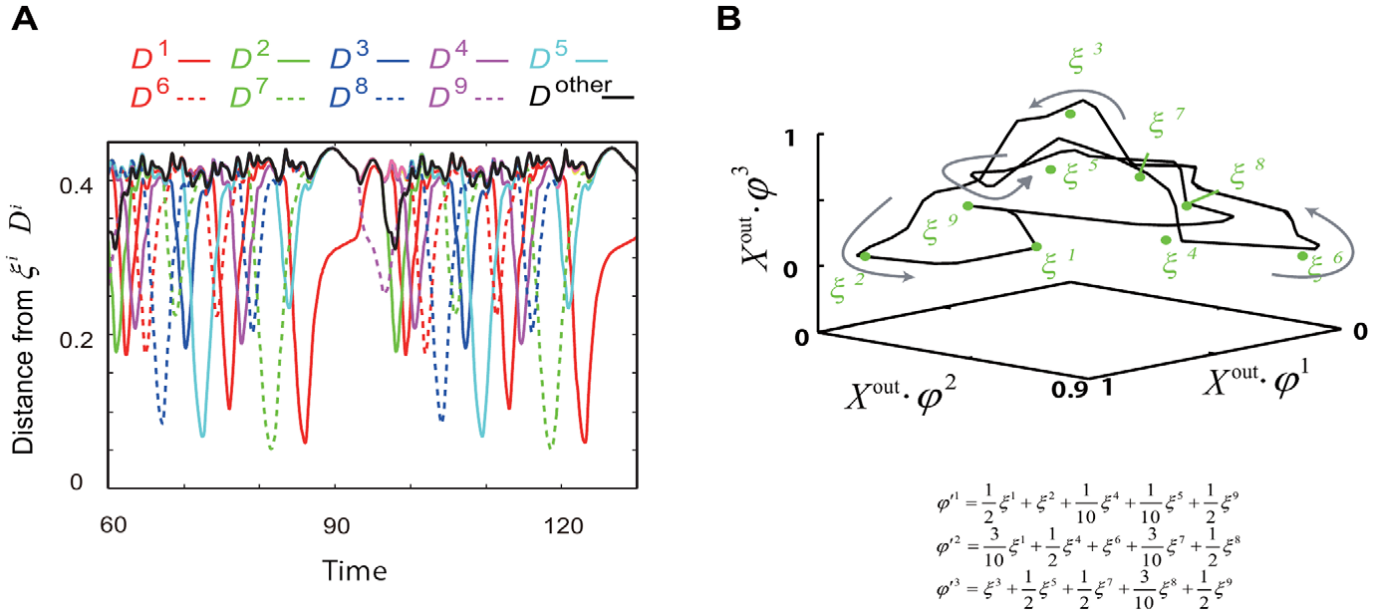
Temporal evolution of neural activity when the attractor without inputs is a complex limit cycle. The spontaneous neural dynamics and the evoked one after learning nine I/O mappings are plotted, same as Figs. 7 and 8. A) The time courses of the distance between the neural activity of the output and each target are plotted as Fig. 8. B) An example of complex spontaneous activity dynamics. Neural activity in the output layer is represented as Fig. 8. Each axis represents the product of the neural activity and the corresponding combined target patterns 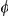, defined in the figure. The plot shows the locus of neural activity, which yields a complex limit cycle in the absence of inputs. Green circles represent the learned targets patterns. The limit cycle approaches some of the targets more than once.

There are two types of modifications in the neural dynamical system in our model: modification through learning (i.e., change in the synapse strength) and that by the injection of the input (i.e., change in the input strength). To understand these modifications, we first analyze the modification of spontaneous dynamics by the learning process and then study the modification upon the injection of an input. The abovementioned modification and analysis are discussed in the present and subsequent subsections, respectively. To be specific, we examine the typical orbits of neural activity in the absence of any input, by considering a dynamical system with fixed synaptic strength in the early and late stages of the learning process. Figures 7, 8, and 9 show examples of typical orbits in the attractors, determined on the basis of the results of the 4th, 8th, and 9th learning steps. After targets 1, 2, 3, and 4 are learned, the neural activity in the output layer in the absence of any input is itinerant over three patterns that are close to three of the target patterns before the neural activity reaches a fixed point, as shown in Figure 7. The output activity approaches targets 3, 4, and 1 successively before converging to the fixed point. This itinerancy over the targets at transient time is commonly observed for several initial conditions in the orbits in the early stages of the learning process.

**Figure 10:**
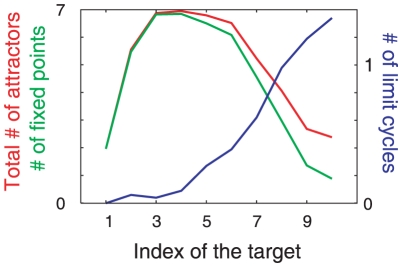
Change in the number of attractors during the learning process. Numbers of fixed-point attractors (green line) and limit-cycle attractors (blue line) and the total number of attractors (red line) in the absence of inputs as a function of the number of learning steps, i.e., number of learned targets, are plotted. In the early stages of the learning process, the number of fixed-point attractors increases, while in the later stage (after 5 or 6 learning steps), the number of fixed-point attractors decreases (fixed-point attractors are replaced by limit-cycle attractors).

As the learning progresses further, two prominent changes are observed in the neural dynamics in the absence of inputs. First, there is an increase in the number of attractors in the system. As shown in Figure 10, the number of fixed-point attractors increases with the number of learned mappings. With a further increase in the number of the learning steps, however, the number of fixed-point attractors begins to decrease, as these attractors are replaced by one or more limit-cycle attractors. A limit cycle, however, does not always appear, but its emergence depends on the earlier learning process.

The limit-cycle orbit itinerates over target patterns as an attractor; in contrast, the orbits in the fixed-point attractor are transient. Figure 8 shows an example of an orbit at a limit-cycle attractor without inputs after eight I/O mappings are learned, such that the orbit (not a transient orbit as shown in Fig. 7) itinerates over the targets in the cyclic order 1, 2, 4, 7, and 5 (12475). Here, we describe a limit cycle itinerating over targets in the order a, b, c (abc) for simplicity. Note that the number of itinerated targets is not always equal to the number of learned targets; further, the order of itineration over the targets is not same as the order of target learning, but depends on each trial of the learning process. For example, after learning five sets, some of the limit-cycle attractors cover all the memorized patterns (12345), while some others cover the memorized patterns only partially (123). In addition, some limit cycles are highly complex and visit the neighbors of the same targets a few times during a given cycle in the order (1345712589), as shown in Figure 9. In this sense, the flow of neural activities in the absence of inputs “prepares” for the target output pattern to be stabilized by the inputs. These itinerant dynamics in the absence of inputs may correspond to the spontaneous activity dynamics in the brain, as will be discussed later.

**Figure 11:**
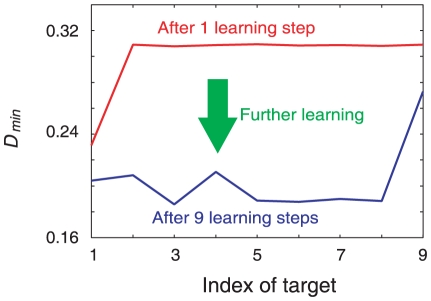
Change in the minimum distance between the activity and the target during the learning process. 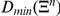
 gives the minimum distance between the neural activity 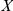 and the target 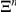, averaged over phase space in the absence of inputs. Here, 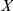 and 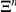 include the activity of the neurons in the output and hidden layers. The target 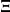 becomes identical to the neural activity pattern after convergence to target 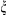 in the output layer. 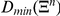 is defined as 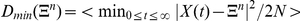, where 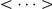 is the average over the initial conditions. The average 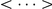 is obtained over 1000 initial conditions for the neural activities 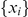. The red and blue lines represent 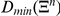 about all targets 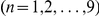 after learning one and nine (I/O) mappings, respectively. Only the minimum distance between the neural activity and the learned target decreases.

In the learning process, the flow structure of the neural dynamics is modified so that the neural dynamics in the absence of inputs come closer to the learned target patterns. We compute the distance between a target and an output neural activity by starting from a given initial condition for the neural activity. Here, we define 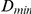 as the minimum distance between the a target and the output activity in the time course, averaged over a variety of initial neural states after each learning step (See caption of Fig. 11). Figure 11 shows the plot of the aforementioned minimum distance versus the number of learning steps. As the learning process progresses, the minimum distance between the output activity and each target learned so far successively decreases. This indicates more orbits from initial points in the phase space come close to the target patterns, after the target patterns are learned. This decrease is observed only when the condition 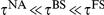 is satisfied. Indeed, when 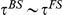 or 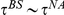, the distance between the output activity and a few latest targets is small, but the distance between the output activity and the earlier targets is large. This observation suggests that traces of previous memories have already been erased (Figure S1).

### Bifurcation with the Input Strength

To close the Results section, we study how the attractor of neural dynamics changes from the attractor for spontaneous activity to that representing the desired output when the input strength is increased. We show that when an input is applied, the attractor of the spontaneous activity dynamics bifurcates into a fixed-point attractor that represents the corresponding target, we also demonstrate, that depending on the input pattern, a distinct attractor corresponding to each target pattern is generated.

**Figure 12:**
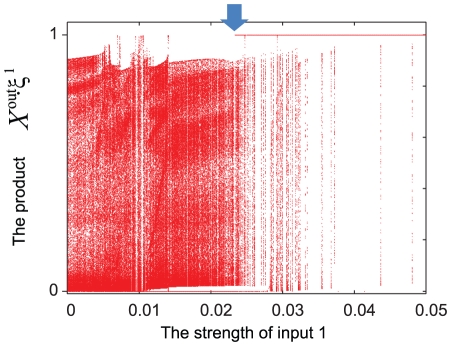
Bifurcation diagram through the input strength. The bifurcation diagram from the spontaneous limit cycle to the fixed point matching the target pattern is shown. The x-axis and the y-axis represent the strength of input, here, input 1 in Fig. 8, and the product between the neural activity in the output layer and the corresponding target. At each strength, we compute the neural activity evolving from an initial point chosen randomly and measure the product between the neural activity and the corresponding target for some constant time after transient time. Points representing values of this product are super-positioned at each strength. In the strength at more than 0.23 (see the arrow), the target is stable fixed-point attractor. In this area, both target fixed point and other attractors are stable, and at larger strength, only the former is stable, means this target is memorized in this network.

Examples of such changes are shown in Figures 7, 8, and 9, where the attractor without inputs is a fixed point or a limit cycle. When an input is applied and its strength is increased, bifurcation occurs such that the original attractor (without the input) becomes unstable, and a stable fixed point representing the correspondent target pattern emerges. To carry out bifurcation analysis, we vary the input strength 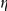 continuously instead of choosing the large input value adopted in the learning process, and study the corresponding changes in the attractor. When the spontaneous attractor is a fixed point, it remains stable up to a certain value of 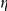. With an increase in 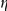 beyond the threshold, saddle-node bifurcation to a novel fixed-point attractor occurs. When the attractor in the absence of inputs is a limit cycle, it collapses as a result of non-local bifurcation, and is replaced by the fixed-point attractor corresponding to each target pattern, with an increase in 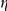. An example of a bifurcation diagram from the limit cycle to the fixed point for the target output pattern is shown in Fig. 12, where the activity of the target neuron at the attractor is plotted as a function of 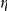. As shown, the changes in the attractor involve several bifurcations, and the whole bifurcation sequence becomes complicated with an increase in 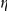. Nevertheless, there are two common characteristics: collapse of the limit cycle for the spontaneous activity, as a result of non-local bifurcation and appearance of the target fixed point by saddle-node bifurcation. The former is caused by the contact formed between the limit-cycle attractor and the basin of another fixed-point attractor upon an increase in 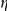, while the limit-cycle and the fixed-point attractors coexist over a certain range of input values, implying hysteresis. The fixed-point attractor generated by this collapse is generally not a fixed point that gives rise to the target output. With a further increase in the input parameters, saddle-node bifurcation leads to the attractor corresponding to the target.

**Figure 13:**
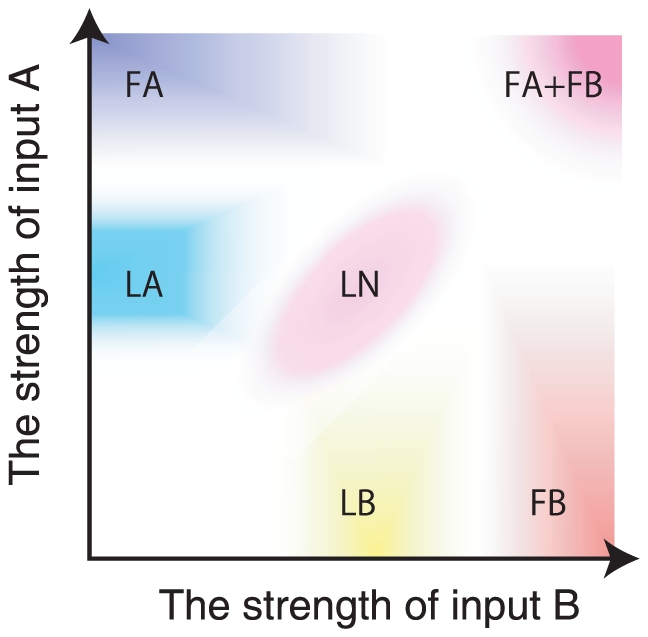
Phase diagram of the neural dynamics under the application of two inputs. After the model neural network learned several input-output relations, we applied the two learned inputs A and B with the strength 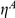 and 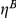. By varying these two input strengths, we checked what type of attractors the neural dynamics are attracted to. Depending on the type of attractor, rough phase diagram is depicted. Each of the phases in the figure represents the following behavior; FA and FB: Only the fixed-point attractor with the output matching with the target A or B (FA) and target B (FB), respectively. FA+FB: Coexistence of the two fixed point attractors for the target A and B, reached depending on the initial condition. LA and LB: Limit-cycle attractor in which the neural dynamics approach the target A or target B, respectively. LN: novel limit cycle attractor in which the neural dynamics approach neither the target A nor target B. Shown here is a rough diagram, and actual bifurcations are more complicated, and also depend on each learning process.

It is also interesting to study the neural dynamics when two learned inputs are injected simultaneously. By changing the strength of each input, bifurcation against two parameters is studied (Fig. 13). We find some characteristic neural dynamics with different input strength. If the strength of input A 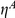 is much larger than that of input B 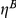, the fixed-point attractor giving rise to the target A (phase FA in Fig. 13) is generated, and vice versa. When 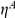 is smaller, but much larger than 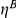, the limit-cycle attractor in which the neural dynamics approaches the target A (phase LA in Fig. 13) is generated, and vice versa. Furthermore, when both 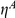 and 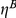 are much large and of the same order, there appears a new phase in which the two fixed-point attractors matching target A and target B coexist. In this case, depending on the initial state, either of targets A or B is retrieved as an output. On the other hand, when both 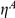 and 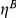 are smaller, novel limit-cycle attractors in which the neural dynamics approaches neither target A nor target B are generated. Since the bifurcations by applied two inputs are complicated, it is not so easy to draw the whole bifurcation diagram; Figure 13 is a rough sketch of the diagram. Detailed study on the bifurcations will be left for future.

### Possible Extension to Learn Complex Mappings

In the present study, we have focused on the learning of one-to-one mapping between inputs and outputs. The strength of synaptic connections turns to be either very strong or very weak after learning, and therefore, only simple one-to-one mapping is possible. However, our model can be extended to allow for complex mapping. By adding more neurons in the hidden and output layers and also by introducing the upper thresholds for synaptic strength, complex tasks in which there is as certain degree of overlap of neurons between different input patterns can be learned and memorized. For example, we have confirmed that our algorithm can learn the following task, when the abovementioned modification is made to the model. The learning task involved mapping on the basis of input patterns (1, 0), (0, 1), and (1, 1) in the input layer, while the corresponding target patterns are set as (1, 0, 0), (0, 1, 0), and (0, 0, 1) respectively. Even though tuning the upper boundary of the synaptic connections is required, we have confirmed numerically that memorizing these I/O relationships with the overlapped inputs is indeed possible. Learning more complex tasks can be made possible by appropriate adjustment of the synaptic strength. We expect that our viewpoint -learning and memorizing of I/O patterns by shaping the neural activity dynamics so that they undergo the appropriate bifurcations- can be applied to more complex cases by appropriate extension of the present model.

## Discussion

In the present paper, we have proposed a novel image of memory by using a dynamical system model for I/O mapping. Memory recall is achieved as a result of the bifurcation of the neural dynamics attractor from a spontaneous activity attractor to one that matches the target pattern induced by the input. The learning process shapes the “appropriate” flow structure of spontaneous neural dynamics. Indeed, this bifurcation viewpoint is consistent with the change in neural activity observed in a olfactory system in the recent experiment [6], which has been discussed in the Introduction.

To demonstrate the learning process on the basis of the aforementioned idea, we present a model in which the learning process shapes the flow structure of the neural dynamics, through successive presentations of inputs and the corresponding outputs. We use a three-layered network with forward synapses from an input layer to a hidden layer and from the hidden layer to the output layer, as well as backward synapses from the output layer to a hidden layer; the use of such a network allows for autonomous neural dynamics even in the absence of inputs. Such an architecture with backward connections has been adopted for SRNs [10] [11], echo state networks [13], and liquid-state machines [14]. The neurons in the hidden layer in these models are activated not only by the input neurons but also by the neighboring neurons in the layer; consequently, the dynamics of these neuron activities are not determined solely by the inputs but can autonomously change even in the absence of inputs, as in the case of our model. Studies on these models, however, have focused mainly on responses against input streams, with the aim of analyzing temporal information processing on the basis of the input history. In contrast, our study focuses on the change (bifurcation) from spontaneous activity dynamics to evoked dynamics in the presence of inputs and on the shaping of spontaneous dynamics through the learning process.

For analyzing the changes in the flow structure during the learning process, the following points are discussed. First, to achieve the maximum number of memorized patterns, the appropriate relationship has to be satisfied among the time scales of the changes in the neural activity as well as among those of the changes in the plasticity of the FSs and BSs. Second, the flow structure of the spontaneous dynamics is changed during the learning process, and then, the neural dynamics in this flow structure are itinerant over the learned output patterns. In other words, spontaneous dynamics “prepare” for the stabilization of the corresponding outputs once the inputs are provided. Now, we discuss these two points in possible relationship with the results of recent experimental studies.

### Synaptic Plasticity

We adopt two architectures for synaptic plasticity: (i) multiple timescales and (ii) the ARP algorithm. Here, we discuss how these architectures can be implemented in our brain.

#### (i) Multiple time scales

The time scale of synaptic dynamics represents the magnitude of the synaptic plasticity, such as long-term potentiation and long-term depression, and these plasticities depend on the number and/or type of receptors of neural transmitters in our brain. Hence, the time scales for synaptic plasticity are related to the number and/or type of these receptors. When two areas (say between the hippocampus and the prefrontal cortex) are mutually connected, the synaptic connections for forward and backward connections may have different characteristics, and hence, the plasticities may differ between the two.

Recall that in our model, a proper relationship has to be satisfied among the time scales of the changes in the FSs and BSs and in the neural activity, to achieve the maximum number of memorized patterns. On the basis of the above argument, it is suggested that such a difference in the plasticities may be implemented by the possible difference between the number distribution and/or types of receptors in the neurons for the forward and backward connections between the given areas.

#### (ii) Adaptive reward-penalty

In our model, the synaptic plasticity is switched between Hebbian and anti-Hebbian rules by the ARP algorithm, depending on the magnitude of the error signal. In our brain, neural modulators such as dopamine, serotonin, norepinephrine, and acetylcholine may give rise to this error signal. In particular, dopamine modulates the synaptic plasticity at the hetero-synaptic connection [15] and is projected onto the cerebral cortex broadly. Furthermore, the activity of dopamine neurons is related to the extent to which the response matches the request [16]. Hence, dopamine can act as a global error signal carrier. Then, it is suggested that the switching between positive and negative plasticity, corresponding to that between the Hebbian and anti-Hebbian rules in our model, is regulated by the concentration of dopamine.

In the present study, we show that the maximum capacity can be realized by establishing an appropriate relationship between the time scales of FS and BS plasticity, and the results suggest that such multiple timescales would be important for memorizing. Recently, Fusi et al. [17] proposed a meta-plasticity-based model, which may involve multiple timescales similar to in our model, In their model, while the synaptic plasticity changes with external stimuli, the change is only stochastic, and neither neural activity dynamics nor synaptic plasticity is considered. In contrast, in our model, the interaction between neural dynamics and synaptic plasticity plays a key role in the memory process.

### Spontaneous Activity

Next, we discuss the spontaneous activity in our model and experimental studies. Recent experimental studies have revealed the existence of spontaneous neural activity, which is not simply noise but a result of the inherent dynamics in the brain [5] [9] [18] [19]. Furthermore, such activity is shown to be related to the task-evoked or stimulus-evoked activity [9] [18] [19]. In particular, it has been stated that the spontaneous activity in the visual system of a cat successively changes from one pattern to another [9]. Remarkably, these patterns are found to correspond to those evoked by a directional input signal. These evoked patterns are formed depending on the environment after birth, i.e., these patterns are formed by learning. Thus, the spontaneous dynamics itinerating over the learned patterns are shaped in the manner shown in our model, in which the target pattern is considered to be identical to the pattern evoked by visual stimuli.

Theoretical discussions on such itinerant dynamics of neural activities have been carried out, with attractor ruin networks [20] [21] and heteroclinic cycles [22] [23]; experimental evidence for these dynamics has also been provided [24]. In these studies, it has been show that the neural activity itinerates over the memorized patterns one by one, similar to the spontaneous activities in our model. However, the manner in which learning gives rise to these itinerant dynamics has not been clarified. In our study, we show the generation of spontaneous activities by the successive learning of I/O mappings through Hebbian and anti-Hebbian rules.

We put forward the idea “memory as organization of flow structure” or “memory as flow,” which is in sharp contrast to the idea of “memories as attractors.” According to proposed idea, the neural dynamics in the presence and absence of different inputs are distinct and separated because of the change in the flow of neural activities. The distinct change in the flow is formulated as bifurcation, which stabilizes distinct memorized patterns (in this sense, our viewpoint can also be termed “memories as bifurcations”). This bifurcation against the input strength will be experimentally confirmed by measuring the neural activity for different external stimuli.

## Supporting Information

Figure S1
**Change in the minimum distance between the activity and the target.** Change in the minimum distance between the output activity and the target during the learning process are plotted. The minimum distance *D_min_* at 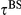 = 1 and 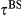 = 128 is plotted in Figs. A and B, respectively. Here, *D_min_* is defined in the same manner as in Figure 9. Red and blue lines represent *D_min_* after one and ten learning steps, respectively. The distance to one (or a few) target(s) is small, while in Figure 9, the distance to almost all the targets is small for 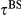 = 8.(PDF)
