# Supplementary material for: Learning Shapes Spontaneous Activity Itinerating over Memorized States
Source: PLoS One. 2011 Mar 8;6(3):e17432. doi: 10.1371/journal.pone.0017432 (PMC3050897; doi:10.1371/journal.pone.0017432)
Supplement: Figure S1 — Change in the minimum distance between the activity and the target. Change in the minimum distance between the output activity and the target during the learning process are plotted. The minimum distance Dmin at = 1 and = 128 is plotted in Figs. A and B, respectively. Here, Dmin is defined in the same manner as in Figure 9. Red and blue lines represent Dmin after one and ten learning steps, respectively. The distance to one (or a few) target(s) is small, while in Figure 9, the distance to almost all the targets is small for = 8. (PDF) [file pone.0017432.s001.pdf]

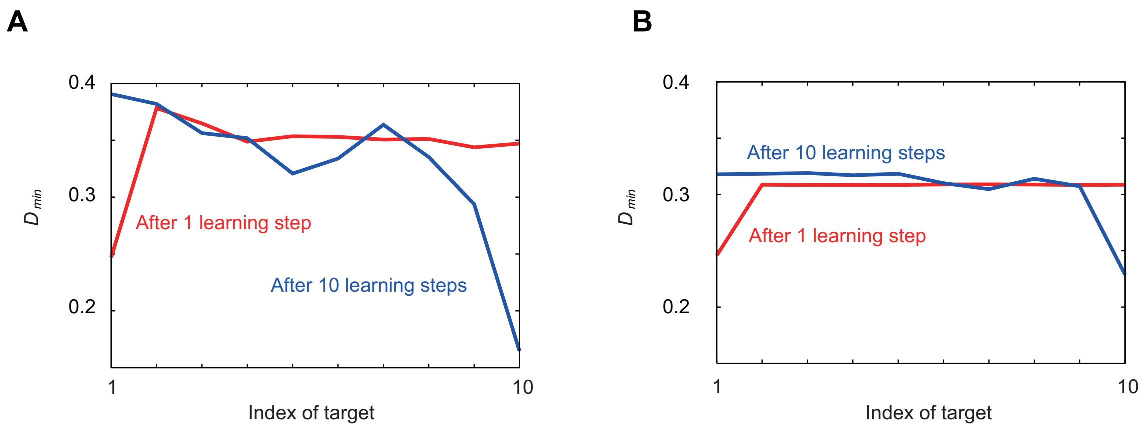

A
0.4
0.3
0.2
D_{min}
After 1 learning step
After 10 learning steps
1
10
Index of target
B
0.4
0.3
0.2
D_{min}
After 10 learning steps
After 1 learning step
1
10
Index of target

Supporting Figure Legends

Figure S : Change in the minimum distance between the activity and the target. Change in the minimum distance between the output activity and the target during the learning process are plotted. The minimum distance D_{min} at $\tau^{BS} = 1$ and $\tau^{BS} = 128$ is plotted in Figs. A and B, respectively. Here, D_{min} is defined in the same manner as in Figure 9. Red and blue lines represent D_{min} after one and ten learning steps, respectively. The distance to one (or a few) target(s) is small, while in Figure 9, the distance to almost all the targets is small for $\tau^{BS} = 8$.
